# Time-delay reservoir for signal demixing using Kalman weight updates in fixed point and limit cycle regimes

**DOI:** 10.1038/s41598-026-38398-7

**Published:** 2026-02-11

**Authors:** S. Kamyar Tavakoli, Jérémie Lefebvre, André Longtin

**Affiliations:** 1 Department of Physics, University of Ottawa, Ottawa, Ontario K1N 6N5 Canada; 2 Krembil Brain Institute, University Health Network, Toronto, Ontario M5T 0S8 Canada; 3 Department of Biology, University of Ottawa, Ottawa, Ontario K1N 6N5 Canada; 4 Department of Mathematics, University of Toronto, Toronto, Ontario M5S 2E4 Canada; 5 Center for Neural Dynamics and AI, University of Ottawa, Ottawa, Ontario K1N 6N5 Canada

**Keywords:** Engineering, Mathematics and computing, Physics

## Abstract

In this paper, we study the problem of separating chaotic signals using time-delay reservoir computers with online training via Kalman filtering. Time delay reservoir computers are hardware-efficient and suitable for experimental, high-speed implementation. We demonstrate that incorporating an online training scheme significantly enhances the performance of time-delay reservoirs in challenging signal demixing tasks. In particular, we apply a sliding-window technique to update the readout weights and show that it can improve accuracy compared to the offline ridge regression readout in various scenarios. Here we mainly focus on the separation of two trajectories generated by the Lorenz system with different initial conditions, which is an especially difficult task since both signals share nearly identical statistical properties. We also study mixtures of signals from two different systems, specifically the Lorenz and Mackey–Glass systems, to predict the signal that contributes weakly to the mixture. Furthermore, this approach enables the time-delay reservoir computer to operate effectively in regimes where the nonlinear delay differential equation exhibits a limit cycle attractor in the absence of input, which we find to be less affected by small inaccuracies in the online weight updates than the stable fixed-point regime. This broadens the range of dynamical settings suitable for signal separation. The highest prediction accuracy, regardless of window size, is typically achieved near critical points where the system’s qualitative behavior changes.

## Introduction

Signal separation, or recovering individual sources from mixed observations, is a fundamental challenge across domains such as neuroscience and communications. In real-world signal processing, observed data typically consist of mixtures of multiple sources rather than isolated components. The brain can selectively attend to one speaker when multiple voices are present, a situation often referred to as the cocktail party problem, in which listeners focus on a specific speaker amidst background speech^1,2^. Although the brain performs this selective processing effectively, its underlying mechanisms remain under investigation. Feedback-based cancellation has also been proposed in studies of weakly electric fish, where a global feedback mechanism enables the cancellation of predictable stimuli and enhances the detection of novel stimuli^3^.

Signal separation is crucial to extracting meaningful information from complex measurements in many scientific and engineering domains. In electroencephalography (EEG), for instance, isolating seizure activity from artifacts and noise is important for accurate seizure detection and monitoring^4^. Computational tools include higher-order statistical methods such as independent component analysis^5^, as well as methods based on time-delayed correlations^6^ and deep learning techniques^7^.

Echo-state networks (ESNs) are reservoir computing models with a fixed high-dimensional recurrent layer (the reservoir) that nonlinearly transforms the input, while only a linear readout is trained. This compact training scheme scales well and has been applied to tasks ranging from speech recognition^8^ to chaotic time-series prediction^9,10^, and robotics tasks^11^. Remarkably, a delayed ESN trained on a single time series at one fixed delay value can predict dynamics for other values of the delay parameter by changing the ESN’s internal delay after training^12^. Reservoir computing models have also been shown to perform source separation. For instance, Krishnagopal et al. demonstrated that an ESN can separate a mixture of two Lorenz trajectories^13^. The reservoir’s large, nonlinear state space can magnify subtle differences between signals, making them more nearly linearly separable by the readout. In contrast, classical ICA is not expected to work reliably here because ICA relies on statistical independence assumptions, whereas signals drawn from the same chaotic system can have similar marginal distributions and exhibit dependencies that can reduce ICA identifiability. By leveraging the ESN’s transient dynamics and memory, however, it can be possible to disentangle even subtly different chaotic trajectories. This challenge has also been addressed using Time-Delay Reservoir Computing(TDRC)^14^, where single-node architectures have successfully separated mixtures of distinct chaotic systems sampled at different rates and disentangled chaotic signals embedded in correlated noise. Beyond signal demixing, TDRC has been applied both computationally and experimentally for signal classification and speech recognition^15–18^.

For single-signal prediction, performance can depend on different dynamical properties; for example, limit cycle operation can enhance computational performance in time-delay reservoir computing^19^. Furthermore, improvements have been achieved through architectural innovations: hierarchical reservoirs and deep multilayer structures reduce prediction error^20,21^, while additional delay lines enhance memory capacity and accuracy^22^. Interestingly, a single nonlinear node with multiple delayed states can realize deep feedforward and recurrent topologies for arbitrary networks via time-unfolding^23^. In multi-delay systems, few delays can increase entropy depending on the system and the spacing between delays, whereas broadly distributed delays may reduce complexity^24,25^.

Nevertheless, a critical limitation persists when conventional TDRC encounters signal mixtures in which a dominant component with strong autocorrelation masks a weaker chaotic source. This occurs, for example, in the mixture of Mackey–Glass (MG) and Lorenz *x*, where the MG signal can dominate or obscure the less correlated Lorenz *x*, depending on the mixing ratio. A similar challenge arises in the mixture of two Lorenz *x* signals with different initial conditions or slightly different parameters. In this case, both components share similar autocorrelation structures, making it difficult to distinguish them based on second-order statistics alone. In both cases, we explore a full range of mixing ratios ($s \in [0,1]$), for scenarios where one source is dominant, balanced, or weak. The present work addresses this limitation by implementing online training of the readout weights via a Kalman filter ^26^, extended here to incorporate a sliding-window update scheme. This enables reliable recovery of concealed chaotic components while maintaining the efficiency of single-delay reservoir architectures.

## Methods

### Time–delay reservoir computer

We consider two chaotic signals, $u_1(n)$ and $u_2(n)$, either generated from two Lorenz systems with identical parameters but different initial conditions or from two distinct sources such as the MG and Lorenz systems. These signals are mixed according to

1 $$\begin{aligned} u(n) = \sqrt{s} \, u_1(n) + \sqrt{1 - s} \, u_2(n), \end{aligned}$$

where $s \in [0, 1]$ controls the relative contribution of each component. Each of the signals $u_1 (n)$ and $u_2 (n)$ is normalized to have zero mean and unit variance. The mixed signal *u*(*n*) is then used as input to a TDRC, with the goal of recovering one of the source signals. The target output is defined as $O_2(n) = u_2(n+1)$ when recovering the second source, and analogously $O_1(n) = u_1(n+1)$ for the first source. A scalar, pre-processed input sequence *u*(*n*) is held constant over each clock cycle of duration *T*. To create a high-dimensional state space analogous to a reservoir computing’s dynamics, the input is modulated by a binary mask $m(t) \in \{-1, +1\}$. The mask is piecewise constant over shorter intervals $\theta$, defined by

2 $$\begin{aligned} m(t) = m_i \in \{-1, +1\}, \qquad t\in \big [nT+i\theta ,\; nT+(i+1)\theta \big ),\ \ i=0,\dots ,N_v{-}1. \end{aligned}$$

where $\theta = T/N_v$ is the spacing between virtual nodes, and $N_v$ is the number of virtual nodes per cycle. The resulting continuous-time driving signal becomes

3 $$\begin{aligned} J(t)=u(n)\;m(t)\quad \text {for}\quad t\in \big [nT+i\theta ,\; nT+(i+1)\theta \big ). \end{aligned}$$

The masked input *J*(*t*), defined above, drives the dynamics of the system and causes rich transient responses. The electro-optic oscillator with external input evolves according to:

4 $$\begin{aligned} \dot{x}(t)&= -x(t) + \beta \sin ^2\left( x(t - \tau ) + \phi + \gamma J(t)\right) - y(t), \end{aligned}$$

5 $$\begin{aligned} \dot{y}(t)&= x(t), \end{aligned}$$

where $\beta$ is the feedback gain, $\gamma$ is the input scaling factor, $\phi$ is a phase offset, and $\tau$ is the delay time, which governs the dynamics of the system by determining how past states influence the current state. This differs from the clock cycle *T*, which determines how long each input sample is held constant and defines the sampling steps within that period. Unless otherwise stated, parameter values are listed in Table 1. As established in prior works on TDRCs,  absence of input typically leads the dynamics to a stable fixed point. This stable fixed-point regime is typically associated with fading memory and with behaviour analogous to the echo state property in reservoir computing. The node state *x*(*t*) is sampled at $N_v$ equally spaced virtual nodes per cycle, defined at times $t_{n,i} = nT + i\theta$ for $i = 0, \dots , N_v{-}1$. The resulting reservoir state vector at step *n* is:

6 $$\begin{aligned} \textbf{x}(n) = \bigl [x(t_{n,0}),\, x(t_{n,1}),\, \dots ,\, x(t_{n,N_v{-}1})\bigr ]= \bigl [x(nT),\, x(nT + \theta ),\, \dots ,\, x(nT + (N_v{-}1)\theta )\bigr ]. \end{aligned}$$

Let $X_{M \times N_v}$ be the matrix formed by stacking reservoir states, with *M* being the number of datapoints (for example, $M = M_{\text {train}}$ during training or $M = M_{\text {test}}$ during testing):

$$X = \begin{bmatrix} \textbf{x}(1) \\ \textbf{x}(2) \\ \vdots \\ \textbf{x}(M) \end{bmatrix},$$

Following this, a standard ridge regression can be applied to train a linear readout. For signal separation, two independent regression problems are formulated using the target signals $O_1$ and $O_2$, where each row of $O_i$ corresponds to the desired output at time step *n*. The readout weights $W_{1,2}$ are obtained by solving:

7 $$\begin{aligned} & W_1 = (X^T X + \lambda \textbf{I})^{-1} X^T O_1, \end{aligned}$$

8 $$\begin{aligned} & W_2 = (X^T X + \lambda \textbf{I})^{-1} X^T O_2, \end{aligned}$$

where $\lambda$ is a regularization parameter and $\textbf{I}$ is the identity matrix. So this is the process of offline training where two prediction outputs can be obtained:

9 $$\begin{aligned} & \hat{O}_1=X W_1 \end{aligned}$$

10 $$\begin{aligned} & \hat{O}_2=X W_2 \end{aligned}$$

**Table 1:** Simulation parameters.

Symbol	Description	Value
$M_{\text {train}}$	Training size	20,000
$N_v$	Number of virtual nodes	50
T	Clock cycle	40
$\tau$	Time delay	40
$\phi$	Phase offset	$2\pi /5$
$\lambda$	Regularization parameter	$10^{-6}$
$\gamma$	Input scaling (fixed point)	0.135
$\gamma$	Input scaling (limit cycle)	0.035
$\beta$	Feedback gain (fixed point)	1.66
$\beta$	Feedback gain (limit cycle)	2.38

### Kalman read-out adaptation

In this section, we describe the Kalman filtering method for adaptive readout training in time-delay reservoir computing, as introduced in ref.^26^. Kalman filtering has previously been used for blind source separation in state-space models^27^. Once the reservoir state $\textbf{x}(n)$ is sampled at each clock cycle, the read-out weights $W_{1,2}$ are updated recursively. The recursion is initialized from the offline ridge solution:

11 $$\begin{aligned} W_{1,2}^0= W_{1,2}^{\text {ridge}} \end{aligned}$$

This recursive weight update enables online learning by continually adjusting the weights based on the most recent prediction error. At each step, the predicted output is given by

12 $$\begin{aligned} \hat{y}_{1,2}(n)=\textbf{x}(n)W_{1,2}^{n-1}. \end{aligned}$$

where $\hat{y}_{1,2}(n)$ is the estimate of the target $y_{1,2}(n)=O_{1,2}(n)$ and $W_{1,2}^{n-1}$ is the previous estimate of the weights. In this method, there is uncertainty about the current weight values, which is captured by the covariance matrix *P*(*n*) of the weight estimate $W_{1,2}^n$. We initialize this covariance as $P(0)=I$ and the diagonal elements of *P*(*n*) represent the variances of individual weights, quantifying the uncertainty associated with each weight estimate. Before incorporating the new observation $y_{1,2}(n)$, this uncertainty is updated at each step according to:

13 $$\begin{aligned} P_{-}(n) = P(n-1) + Q\,\textbf{I} \end{aligned}$$

where *Q* is a non-negative scalar that controls how much we add at each step to model possible weight drift. A larger *Q* permits the weights to adapt more rapidly, while $Q \rightarrow 0$ recovers the behavior of static regression. The expected variance of the forthcoming prediction error is

14 $$\begin{aligned} S(n) = R + \textbf{x}(n) P_{-}(n) \textbf{x}^{\!\top }(n), \end{aligned}$$

By tuning the hyperparameters *Q* and *R*, we can control the adaptivity and noise robustness of the system. Balancing these two sources of uncertainty yields the Kalman gain:

15 $$\begin{aligned} K(n)=P_{-}(n)\textbf{x}^{\!\top }(n)\,S^{-1}(n), \end{aligned}$$

which shrinks when *S*(*n*) is large, and increases when confidence in the prediction is high. Weights and covariance are corrected via

16 $$\begin{aligned} W_{1,2}^{\,n}&= W_{1,2}^{\,n-1} + K(n)\bigl [y_{1,2}(n)-\widehat{y}_{1,2}(n)\bigr ],\end{aligned}$$

17 $$\begin{aligned} P(n)&=\bigl [\textbf{I}- K(n)\textbf{x}(n)\bigr ]\,P_{-}(n). \end{aligned}$$

so the initial prediction matches the one that would be obtained by ridge regression, after which the Kalman updates refine the weights online. Adding $Q\textbf{I}$ at every step gives the read-out the flexibility to track gradual changes in the signal mixture that an offline training cannot capture. To exploit the correlation between recent samples, which Kalman filtering can enable, we replace the single-sample update with a block update that assimilates the last $n_w$ observations at once. This is done by defining stacked matrices that aggregate the past $n_w$ samples of the reservoir states and targets:

$$\textbf{X}^{(n)} = \begin{bmatrix} \textbf{x}(n - n_w + 1) \\  \vdots \\  \textbf{x}(n) \end{bmatrix}\!, \qquad \textbf{y}_{1,2}^{(n)} = \begin{bmatrix} y_{1,2}(n - n_w + 1) \\  \vdots \\  y_{1,2}(n) \end{bmatrix}\!.$$

Each row of $\textbf{X}^{(n)}$ corresponds to a past reservoir state vector, and each entry in $\textbf{y}_{1,2}^{(n)}$ is the associated target output value at that time step. In this scheme, although the adaptive training assimilates multiple past steps to update the weights, the prediction task itself remains a one-step ahead prediction.

### Inputs

We considered mixtures of chaotic signals in two distinct scenarios. In the first, the signals are generated from two trajectories of the Lorenz system with slightly different initial conditions. The Lorenz system is defined by the following equations:

18 $$\begin{aligned} \dot{x}= & \sigma (y(t)-x(t)), \end{aligned}$$

19 $$\begin{aligned} \dot{y}= & -x(t)z(t)+\rho x(t)-y(t), \end{aligned}$$

20 $$\begin{aligned} \dot{z}= & x(t)y(t)-b z(t), \end{aligned}$$

with parameters $\sigma = 10$, $\rho = 28$, and $b = 8/3$. We integrate the system using a time step of 0.002 and downsample the *x*-component every 0.05 seconds to generate the Lorenz *x* and $x'$ signals used in the experiments.

In the second case, we study a more heterogeneous mixture, combining the Lorenz *x* signal with a signal generated by the MG delay differential equation:

21 $$\begin{aligned} \frac{dx}{dt} = -a x(t) + \frac{b x(t-\tau _{MG})}{1 + x^{10}(t-\tau _{MG})}, \end{aligned}$$

We use standard parameters $a = 0.1$, $b = 0.2$, and $\tau _{\textrm{MG}} = 17$. The MG signal exhibits strong autocorrelation peaks at specific lags due to its delay-induced structure and has a longer correlation time than the Lorenz *x* signal. We integrate the system using a time step of 0.1 and sample the output every 1 second. While this mixture may not reflect a realistic mixing scenario, our aim is not to simulate a physical mixture but to evaluate how well a mixture of complex input signals can be separated. Although both signals are generated from continuous-time dynamical systems, they are treated here as discrete input sequences to test demixing performance.

## Results

The results for two different scenarios are presented in this section: one involving two Lorenz time series with different initial conditions, and the other involving a mixture of Lorenz and MG signals. These are analyzed across different dynamical regimes. Since the system’s performance strongly depends on the correlation structure and complexity of the input signals, each scenario uses a distinct set of parameters. We first identify optimal parameters for the case of equal contribution from both sources and then examine how varying the mixing ratio affects the system’s dynamics.

### Lorenz-Lorenz

**Fig. 1:**
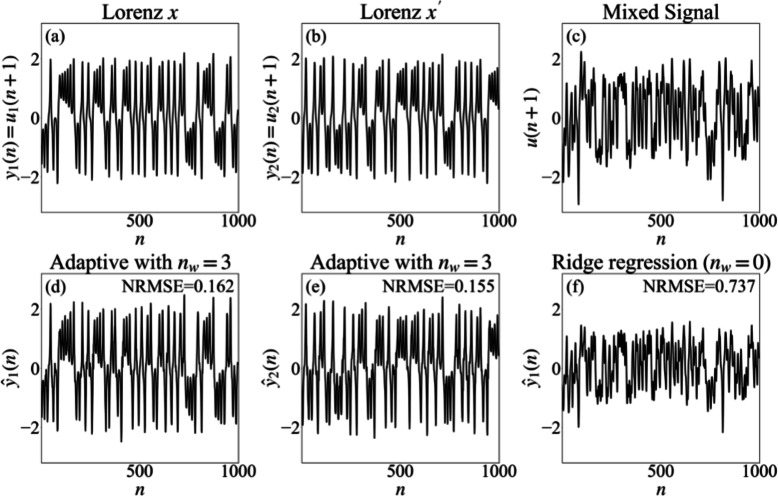
Separation of a mixture of two Lorenz signals using ridge regression and adaptive training. Panel (**a**) presents the Lorenz *x* component $y_1(n)=u_1(n+1)$, while panel (**b**) shows a second Lorenz trajectory $y_2(n)=u_2(n+1)$ generated from nearby initial conditions. The mixed observation $u(n+1)$ for a mixing parameter $s=0.5$ is displayed in panel (**c**). Panels (**d**) and (**e**) contain the predictions $\hat{y}_1(n)$ and $\hat{y}_2(n)$ obtained with an adaptive sliding-window Kalman readout with window size $n_w=3$. Panel (**f**) displays the prediction $\hat{y}_1(n)$ of the Lorenz *x* component obtained with a static linear readout trained by ridge regression $(n_w=0)$. The NRMSE values computed over the plotted interval are reported inside the corresponding panels.

We begin by considering two chaotic time series generated by the Lorenz system with slightly different initial conditions. Although chaotic trajectories are highly sensitive to initial conditions, their long-term statistical properties and attractor structure may be nearly indistinguishable. Figure 1 illustrates representative segments of the Lorenz *x* signal, the Lorenz $x^{\prime }$ signal generated from the same system but with slightly different initial conditions, and their mixture at a balanced mixing ratio $s = 0.5$.

**Fig. 2:**
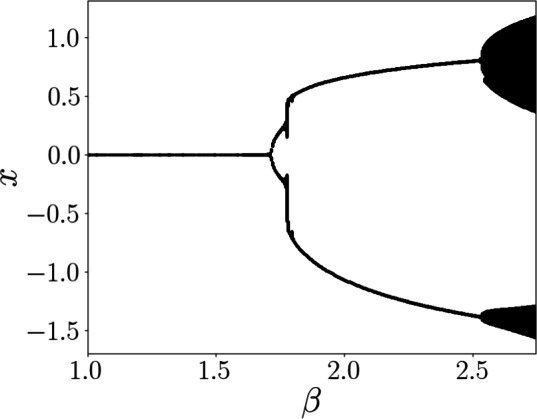
Bifurcation diagram of the electro-optic oscillator model without external input. As $\beta$ increases, the system transitions from a stable fixed point to periodic oscillations near $\beta = 1.68$. At the critical point $\beta = 2.53$, the bifurcation diagram exhibits a broadened range of extrema.

Figure 2 shows the bifurcation diagram of the electro-optic oscillator model in the absence of input, constructed by plotting the local maxima and minima of the state variable *x*(*t*) as a function of the gain parameter $\beta$. For low values of $\beta$, the system converges to a stable fixed point. As $\beta$ increases, a Hopf bifurcation occurs around $\beta = 1.68$, beyond which the system exhibits stable periodic oscillations. The second critical point is close to $\beta =2.53$.

**Fig. 3:**
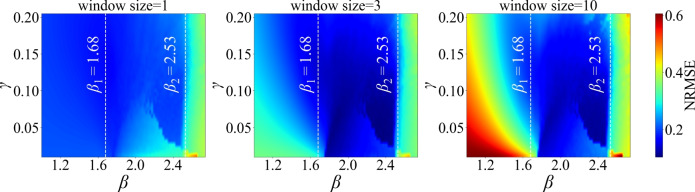
Prediction error versus input scaling $\gamma$ and feedback gain $\beta$ for window sizes of 1, 3, and 10 steps (left to right), using a mixture of two Lorenz *x* signals generated from identical parameters but different initial conditions. Darker colours denote lower error, i.e., higher prediction accuracy. Bifurcations in the underlying reservoir dynamics occur near $\beta = 1.68$ (Hopf bifurcation) and $\beta = 2.53$ (transition to more complex dynamics). Among the three settings, a window size of 3 yields the largest parameter region with consistently low prediction error. In all panels, the Kalman filter hyperparameters are fixed at $Q = 10^{-3}$ and $R = 10^{-4}$.

Prediction accuracy across dynamical regimes is summarized in Fig. 3 using an adaptive Kalman filter that updates the readout weights based on a fixed number of recent data points. Each panel shows the normalized root mean square error (NRMSE) as a function of the feedback gain $\beta$ and the input scaling $\gamma$, with the mixing ratio fixed at $s = 0.5$ for the mixture of two Lorenz *x* signals with different initial conditions. The parameter $\gamma$ modulates the amplitude of the external input *J*(*t*). The three panels correspond to window sizes of 1, 3, and 10 steps, respectively. In all cases, the readout is first trained on $M_{\text {train}}= 20000$ samples and then evaluated on a separate test sequence of $M_{\textrm{test}} = 5000$ points. Lower NRMSE values (darker regions) indicate more accurate prediction. Longer window sizes (multi-step updates) can lead to improved accuracy. However, increasing the window size beyond 3 increases the prediction error in some regions of the parameter space, though accuracy remains higher than with ridge regression or a window size of 1 around the critical points. These improvements are particularly evident near the first and second bifurcation points, corresponding to transitions from a fixed point to a limit cycle and then to a more complex dynamics. This highlights the reservoir’s enhanced computational capacity in regimes of increased complexity. Notably, a window size of 3 yields the broadest region of low error across the parameter space in this example.

**Fig. 4:**
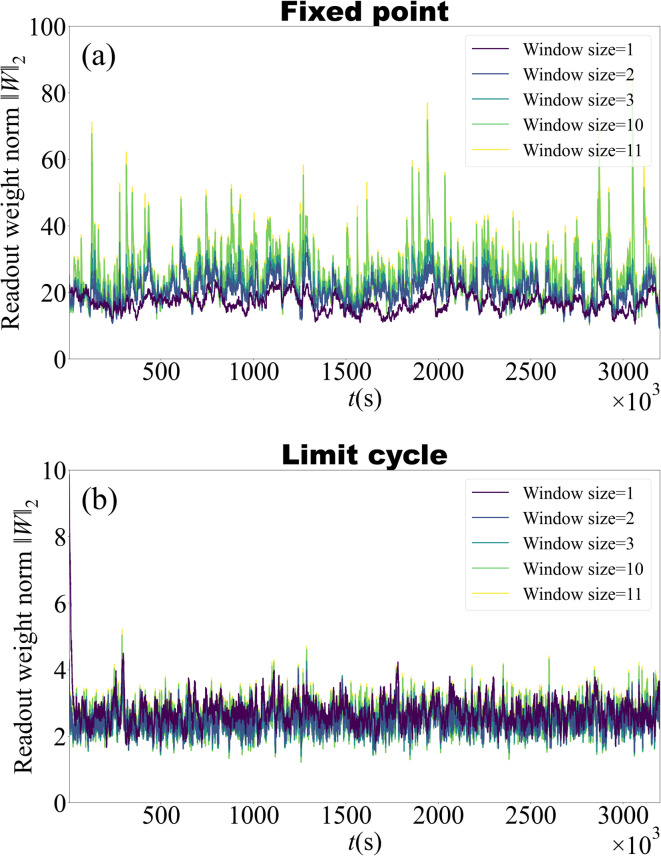
Temporal evolution of the read-out weight norm $\Vert W_{1}^{n} \Vert _{2}$ for different Kalman filter window sizes. **(a)** Stable fixed-point regime. Longer window sizes increase weight fluctuations but keep them bounded. **(b)** Limit cycle regime. Weight norms remain low and change only slightly across all window sizes.

Figure 4 shows the time evolution of the read-out weight norm $\Vert W_{1}^{n} \Vert _{2}$ during online Kalman filter training for window sizes of 1, 2, 3, 10, and 11 samples. The upper panel corresponds to a reservoir operating in a stable fixed-point regime. In this case, the weight norm fluctuates around a baseline, with short-term variability that tends to increase with window size: larger windows are associated with stronger weight fluctuations even in stationary settings. These larger fluctuations may indicate more sensitivity to short-term variations in the prediction error, which may reduce robustness. In contrast, the lower panel shows the system when the parameters correspond to a limit cycle in the absence of input. Here, the weight norm remains roughly one order of magnitude smaller, with fewer and smaller spikes, and exhibits modest, irregular fluctuations of similar variability for all window sizes. This suggests that longer windows can allow adaptation without large increases in variability when the reservoir dynamics are inherently oscillatory.

**Fig. 5:**
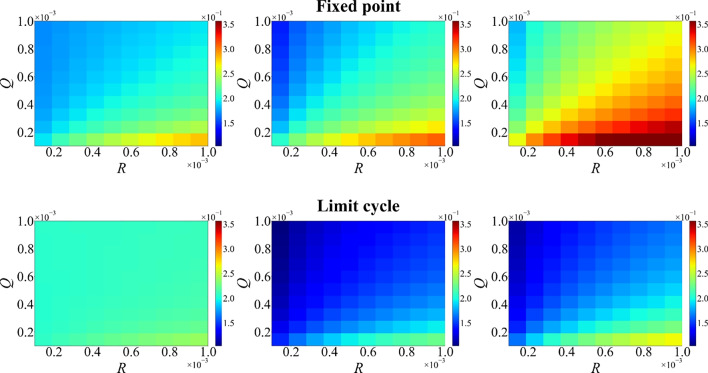
Prediction error as a function of process-noise level  *Q* and measurement-noise variance *R* for a mixing ratio of $s = 0.5$. The top row corresponds to the fixed-point regime, and the bottom row to the limit cycle regime. Columns correspond to window sizes of 1 (left), 3 (middle), and 10 (right) samples. The lowest error occurs when *R* is roughly one order of magnitude smaller than *Q*.

The sensitivity of the Kalman filter to its hyper-parameters is mapped in Fig. 5. The prediction errors are plotted as a function of the process-noise level *Q*, which governs the rate at which uncertainty in the weights is allowed to grow, and the measurement-noise variance *R*, which expresses the assumed uncertainty in the target value *y*(*n*). The lowest prediction error is generally observed when *R* is roughly one order of magnitude (or more) smaller than *Q*; if *R* approaches or exceeds *Q*, the error increases . In the fixed-point regime the low-error region is narrower and is primarily observed when  $R<Q$, whereas in the limit cycle regime it is broader and less sensitive to the exact *Q*/*R* ratio as seen from the larger blue region in the (*Q*, *R*) parameter space, which represents lower prediction error.

**Fig. 6:**
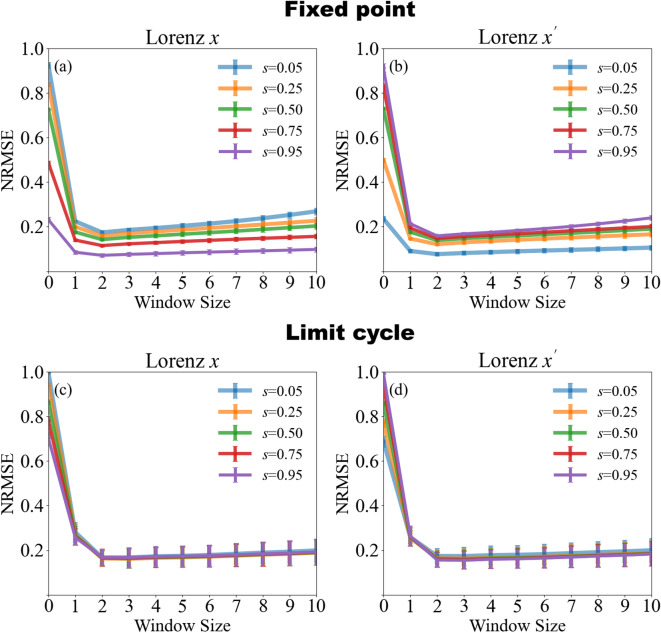
Prediction error as a function of Kalman-filter window size for different mixing ratios *s*. The case where the window size is zero corresponds to the standard ridge regression, i.e. the Kalman filtering is not present. When *s* is large, the Lorenz *x* signal dominates the mixture; when *s* is small, the Lorenz $x'$ signal dominates. **Panels (a) and (b):** reservoir in a stable fixed point. A window of one to three samples minimizes the error for mixed signals, but larger windows increase it. **Panels (c) and (d):** reservoir in a limit cycle regime. The error decreases sharply once the window size exceeds one sample and then levels off, showing little dependence on either window size or mixing ratio. The hyperparameters were set to $Q = 10^{-3}$ and $R = 10^{-4}$, consistent with the setting analyzed in Fig. 5. The limit cycle regime is useful when the target component is weak in the mixture, but outside of those cases, the fixed-point regime generally yields lower prediction error.

Dependence on the window size is explored in Fig. 6 which quantifies how the prediction error depends on the number of recent samples used by the Kalman-filter update (window size, horizontal axis) for several values of the mixing ratio *s*. The upper panels correspond to reservoir parameters that place the system at a stable fixed point, whereas the lower panels show the limit cycle regime. For a window size of 0 (i.e. ordinary ridge regression) the prediction error is low when the target signal is the dominant component of the mixture, but becomes large when the target is weakly represented. In the limit cycle case (lower panels) the NRMSE drops rapidly as soon as one or two additional samples are incorporated and thereafter remains essentially constant, independent of *s*. In the fixed-point case (upper panels) a small window of one or two samples minimizes the error for mixed signals $s > 0$, but larger windows progressively degrade performance; These results indicate that a short window size is sufficient in the periodic regime, whereas the fixed-point regime exhibits an optimal window that balances adaptation. For this task of separating two Lorenz signals with similar statistics, the fixed-point regime yields lower prediction error with short window sizes, while the limit cycle regime offers more consistent performance across window sizes and mixing ratios.

### Lorenz + MG

**Fig. 7:**
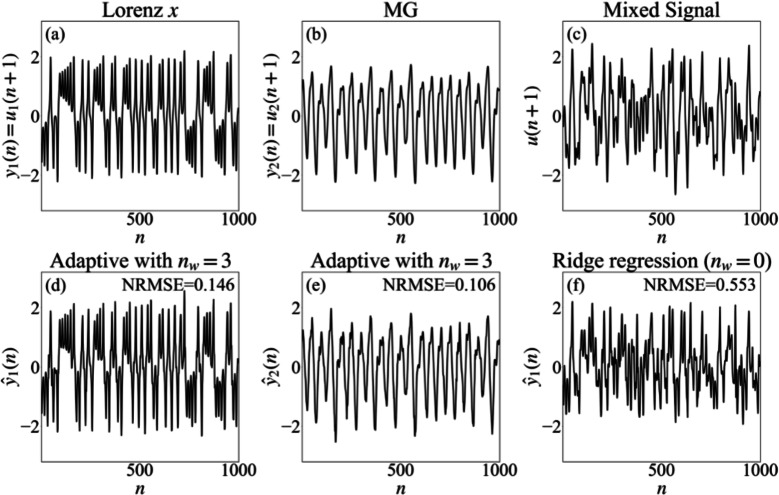
Signal separation of a mixture of Lorenz *x* and MG signals using ridge regression and adaptive readouts. Panels (**a**)–(**c**) show, respectively, the Lorenz *x* component $y_1(n)=u_1(n+1)$, the MG signal $y_2(n)=u_2(n+1)$, and the observed mixture $u(n+1)$ with mixing parameter $s=0.5$. Panels (**d**) and (**e**) display the predictions $\hat{y}_1(n)$ and $\hat{y}_2(n)$ produced by a sliding-window Kalman readout with window size $n_w=3$. Panel (**f**) shows the prediction $\hat{y}_1(n)$ of the Lorenz *x* component obtained from a static linear readout trained using ridge regression $(n_w=0)$. For each predicted signal, the NRMSE between the prediction and its source over the plotted interval is reported inside the corresponding panel.

We also examine the case where the signals originate from two distinct sources: a MG time series and the *x*-component of the Lorenz system. The two signals were generated and sampled at different rates, and representative segments are shown in Fig. 7.

**Fig. 8:**
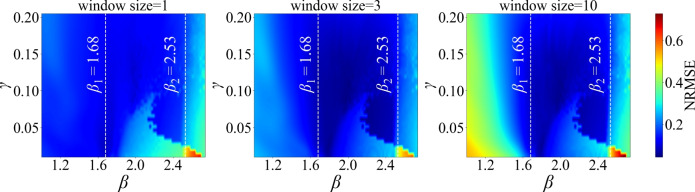
Prediction and separation error for window sizes of 1, 3, and 10, shown across varying feedback gains $\beta$ and input scalings $\gamma$. In the fixed-point regime, the lowest error occurs near $\beta = 1.68$, while in the limit cycle regime it occurs around $\beta = 2.14$. In all cases, the mixing ratio is set to $s = 0.5$, and the Kalman filter hyperparameters are fixed at $Q = 10^{-3}$ and $R = 10^{-4}$.

The prediction error across different input scalings and feedback gains is shown in Fig. 8 for various window sizes. Compared to the same-system mixture (Lorenz–Lorenz) case in Fig. 3, the optimal parameters yielding the lowest prediction error can occur at different values of the input scaling and feedback gain. In the fixed-point regime, the minimum error occurs near the first bifurcation point, close to the onset of oscillations. In the limit cycle regime, the best performance is achieved farther into the limit cycle region, whereas in Fig. 3, it occurs closer to the second critical point.

**Fig. 9:**
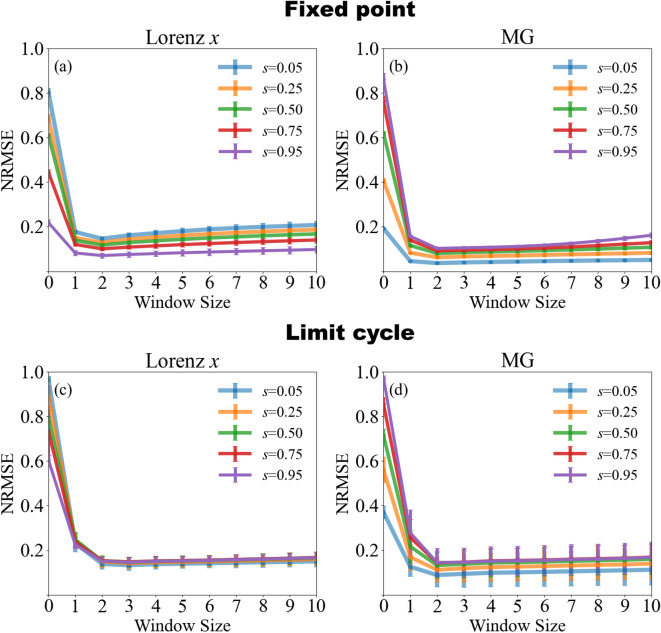
Prediction error as a function of Kalman-filter window size for mixtures of a Lorenz *x* signal and a MG signal sampled at different rates. The case where the window size is zero corresponds to standard ridge regression, i.e., Kalman filtering is not present. When *s* is small, the MG signal dominates the mixture, whereas for large *s*, the Lorenz *x* signal becomes dominant. **(a), (b):** reservoir in a stable fixed-point regime. A window of two to three samples minimizes the error for balanced mixtures ($s = 0.5$), while further increases provide little additional benefit even when one signal dominates. **(c), (d):** reservoir in a limit cycle regime. Prediction improves with window size up to a point, but performance gains are more modest than in the Lorenz *x*+Lorenz $x^{\prime }$ case. These improvements are enabled by the Kalman algorithm, which adapts the readout weights based on recent prediction errors.

It was shown in a multi-delay multi-layer system that when Lorenz *x* dominates the mixture, the structure of the MG signal remains somewhat detectable due to its strong temporal correlations, whereas when MG dominates, the less predictable and weakly correlated Lorenz *x* component becomes much harder to extract ^14^. Introducing online adaptation reduces this imbalance: larger window sizes allow the Kalman filter to track the MG-dominated mixture more effectively, leading to the accuracy gains shown in Fig. 9.  Previously, it was shown that demixing accuracy depends on the mixing ratio ^13,14^. In Fig. 9, we see that increasing the window size using the Kalman technique is especially effective in high-error regimes, such as when the target component is weakly represented in the mixture. In all cases, increasing the window size beyond 2 or 3 steps often yields no further improvement, regardless of the signal.

## Discussion

In this paper, we studied online adaptive training for the separation of complex signal mixtures using time-delay reservoir computing in combination with Kalman filtering with various window sizes. This approach is particularly effective when one aims to extract features that are not easily observable in the raw signal, such as uncovering temporally correlated components that are hidden in the mixture due to the dominance of other signals, or when the two signals in the mixture are from identical chaotic sources sampled from different initial conditions. A representative case is when a Lorenz *x* signal, which lacks clear autocorrelation structure, is contaminated by an MG signal, which exhibits pronounced autocorrelation peaks at specific lags. Although this synthetic example does not directly correspond to a real-world situation, it highlights a fundamental principle: online training enables the readout to adapt continuously, which can improve separation when the mixture components have different temporal structure and uncover informative structure that static readouts may overlook. These results suggest that, even for mixtures of qualitatively different sources, an online adaptive scheme such as updating the weights using the Kalman formalism based on prediction error can substantially improve signal demixing performance.

This type of online training (finite window size) offers enhanced accuracy relative to static learning (zero window size). The main drawback, however, is the need for continuous weight updates, which may introduce computational complexity. Despite this limitation, continuous updating can potentially be beneficial in settings involving nonstationary time series or signals corrupted by noise whose characteristics change over time. In such cases, the ability to track and respond to ongoing shifts in the signal structure may enhance demixing and signal extraction performance.

Predicting the Lorenz *z* signal from a mixture is feasible with good accuracy when the Lorenz *x* component is the dominant contributor, as shown by the fact that Lorenz *x* can be used as the input to predict Lorenz *z* and perform cross prediction using a static linear readout trained by ridge regression^22^. In contrast, attempting to recover Lorenz *x* when Lorenz *z* dominates yields poor accuracy. This asymmetry is related to the correlation properties of the signals, since the autocorrelation of the Lorenz *z* component exhibits distinct peaks at non-zero time lags. Similar behaviour has been shown for mixtures of MG and Lorenz *x*^14^. In this study we considered only scalar inputs taken from time series, but we do not see any conceptual barrier to applying the same approach to scalar observables extracted from more complex spatiotemporal systems such as the Kuramoto–Sivashinsky equation.  Separation of mixed Lorenz-96 and KS signals using a simulated water-tank dynamical system has also been demonstrated^28^, which indicates that such mixtures can be separated using dynamical-systems-based methods.

To enhance the performance of the adaptive training, we examined the effect of incorporating multiple past data points in the weight update process. Increasing the window size initially improves prediction accuracy, but beyond a certain point, excessive reliance on past data leads to larger weight fluctuations. These fluctuations can degrade performance by introducing noise into the weight estimates, ultimately harming the separation quality.

An interesting observation was that, with a single-step update, optimal separation often occurred near the stable fixed-point regime (Figs. 6 and 9). As the window expands to include multiple past steps, the limit cycle regime can yield lower error for certain targets and mixing ratios. In the oscillatory regime, the main predictive power arises from the adaptive Kalman weight updates, which continuously retune the readout to compensate for the tendency of the underlying delayed system to relax back to its limit cycle (where a static readout performs poorly at low input scalings). In fact, the lowest prediction errors are often observed when the system parameters lie near critical points, where the qualitative nature of the dynamics changes. This suggests that the system’s computational capacity is enhanced near bifurcation boundaries. While the absolute prediction error in these regimes may not be as low as in the stable fixed point regime, a notable advantage is that moderately low error can be achieved across a wide range of mixture ratios, highlighting the robustness of the system under varying input conditions.

Interestingly, in the limit cycle regime, the magnitude of variability of the learned weights tends to decrease. This may reflect a greater stability in readout weights, which could be particularly beneficial for offline training or hybrid schemes that combine online Kalman filtering with offline refinement. Smaller weights may also contribute to robustness with respect to hyperparameter choices.

In this paper, we focused exclusively on the effectiveness of Kalman filtering for training time-delay reservoir computers, and did not investigate other important factors that may further enhance performance. For example, the role of the delay parameter in the underlying dynamical system has been extensively studied in the literature, particularly in relation to harmful resonance effects. Incorporating multiple delays or extending the system to a multi-layer architecture may offer additional benefits, which we leave for future work.

## Data Availability

The code used to generate and analyze the results in this study is available from the corresponding author upon request.
